# Comparative analysis of antibiotic susceptibility patterns and clinical features of mucoid and non-mucoid *Pseudomonas aeruginosa* infections: a retrospective study

**DOI:** 10.3389/fpubh.2024.1333477

**Published:** 2024-02-08

**Authors:** Maoling Luo, Si Li, Wenying Luo

**Affiliations:** ^1^Medical Laboratory Center, Affiliated Hospital of Guangdong Medical University, Zhanjiang, Guangdong, China; ^2^General Medicine, Clinical Medicine, Kangda College of Nanjing Medical University, Lianyungang, Jiangsu, China

**Keywords:** *Pseudomonas aeruginosa*, chronic infection, acute infection, risk factors, drug resistance

## Abstract

**Background:**

*Pseudomonas aeruginosa* (PA) is a prevalent opportunistic pathogen that has close associations with both acute and chronic infections. However, there exists an insufficiency of accurate and comprehensive data pertaining to the antimicrobial susceptibility patterns and clinical characteristics of both mucoid and non-mucoid strains of PA (mPA and non-mPA, respectively).

**Methods:**

From January 1, 2021 to December 31, 2022, a thorough retrospective study was carried out to examine and compare the antibiotic susceptibility test outcomes and clinical characteristics of hospitalized patients with mPA and non-mPA infections.

**Results:**

This study investigated a cohort of 111 patients who were diagnosed with mPA infections, as well as 792 patients diagnosed with non-mPA infections. Significant demographic disparities, including gender (*p* < 0.001), age (*p* < 0.001), length of hospital stay (*p* < 0.001), diabetes (*p* = 0.043), and hypertension (*p* < 0.001), are evident between the mPA and non-mPA groups. The mPA group commonly necessitates hospitalization for respiratory system diseases, whereas the non-mPA group is associated with concomitant cardiovascular and cerebrovascular diseases. The mPA group demonstrates lower utilization rates of medical devices, such as Foley catheter (*p* < 0.001), nasogastric tube (*p* < 0.001), mechanical ventilation (*p* < 0.001), tracheostomy (*p* < 0.001), arterial and venous catheterization (*p* < 0.001), and exhibits superior organ function status, including lower incidences of hypoalbuminemia (*p* < 0.001), septic shock (*p* < 0.001), liver dysfunction (*p* < 0.001), renal failure (*p* < 0.001), and respiratory failure (*p* < 0.001). The non-mPA group is more vulnerable to infection with two or more bacterial pathogens compared to the mPA group, with the non-mPA group frequently resulting in Enterobacteriaceae infections and the mPA group being associated with fungal infections. Variations in antibiotic sensitivity are noted for Amikacin (*p* < 0.001), Ciprofloxacin (*p* < 0.001), Cefepime (*p* = 0.003), and Levofloxacin (*p* < 0.001) in antibiotic susceptibility testing, with resistance patterns closely tied to specific antibiotic usage.

**Conclusion:**

There are significant demographic characteristics, clinical manifestations and antibiotic susceptibility between mPA and non-mPA infections. It is crucial to emphasize these characteristics due to their significant role in preventing and treating PA infections.

## Introduction

*Pseudomonas aeruginosa* (PA), a gram-negative opportunistic pathogen, is widely distributed in nature and known for its ability to colonize, adapt, and develop multidrug resistance (1, 2). It possesses a complex and interconnected regulatory system that enables it to thrive in both external and internal environments, often leading to significant morbidity, debilitating diseases, reduced lifespans, and increased mortality rates in humans (3, 4). Through the secretion of various virulence factors, PA exhibits an ability to adapt to hostile host environments, thereby facilitating successful infection and the onset of disease (5). As one of the most common bacteria causing nosocomial infections, it has the potential to induce various infectious conditions, including pneumonia, catheter-related infections, urinary tract infections, wound infections, and bloodstream infections (6). Based on data from the China Antimicrobial Resistance Surveillance System (CARSS) (7), in 2020, the detection rate of PA accounted for 8.66% of all detected bacteria. Additionally, between 2005 and 2017, the prevalence of carbapenem-resistant *Pseudomonas aeruginosa* (CRPA) ranged from approximately 20 to 30%. In the United States, more than 51,000 healthcare-associated infections caused by PA are reported annually, resulting in approximately 440 deaths (8). Approximately 13% (~6,700) of these infections are attributed to multidrug-resistant (MDR) strains of PA. Furthermore, 10–30% of PA isolates demonstrate resistance to carbapenem antibiotics (9). the World Health Organization has designated PA as a “priority pathogen” in urgent need of new antibiotic therapies (10).

The genome of PA demonstrates a remarkable ability to rapidly respond to environmental changes, facilitating the prompt development of adaptive genotypes and the subsequent emergence of new drug resistance mechanisms (11). Non-mPA can undergo mutations in the mucA gene, resulting in the excessive production of alginate exopolysaccharides, a crucial component of biofilms, and leading to a transition to the mucoid phenotype (12, 13). This phenotypic transition, from a non-mucoid to a mucoid state, is accompanied by a shift from an acute virulent phenotype characterized by type III secretion, motility, and toxin production to a chronic virulent phenotype characterized by mucin secretion, biofilm formation, metabolic pathway regulation, alterations in quorum sensing(QS) (14, 15), type VI secretion, and loss of motility (16). During this transition, critical acute virulence genes undergo pathological adaptive functional loss mutations. The expression of various virulence factors associated with acute and chronic infections causes harm to the host, particularly posing a significant threat to critically ill patients with compromised skin mucosal barriers (e.g., severe burns, tracheal intubation, mechanical ventilation) and impaired immune systems (17). This gradual evolution, observed in specific diseases such as cystic fibrosis (*CF*) and chronic obstructive pulmonary disease (COPD), occurs over the course of several decades, progressing from intermittent colonization to chronic infection (18).

The extensive production of extracellular polysaccharide alginate from mPA provides bacteria with a protective barrier against antibiotics. This leads to heightened antibiotic resistance and the potential emergence of multidrug-resistant (MDR) strains, which poses a significant challenge in the field of healthcare (19). Alterations in phenotype and gene expression, coupled with the development of resistance against established antibiotics, reduction in metabolic activity and growth rate, and the production of virulence-associated factors, represent notable characteristics exhibited by biofilm-associated microorganisms (20).

Numerous risk factors for hospital-acquired infections of PA have been identified, including concomitant microbial infections, total parenteral nutrition, coexisting cerebrovascular and cardiovascular diseases, admission to the ICU, malignancies, compromised immune systems, mechanical ventilation, acute respiratory failure, infection sites such as the respiratory tract and central venous catheters, and the utilization of multiple invasive devices (21). Although significant research has been devoted to comprehending the characteristics of the chronic mucoid phenotype associated with PA infections, current literature provides limited clinical data to distinguish individuals with mPA and non-mPA infections, additional clinical validation is required to gain a comprehensive understanding.

In this retrospective study, our aim is to conduct a comprehensive analysis of the demographic characteristics, antimicrobial susceptibility patterns, and infection characteristics associated with mPA and non-mPA isolates. Overall, the integration of various clinical parameters enables a more robust evaluation of PA infections, facilitating a deeper understanding of the disease and supporting evidence-based practices in managing and preventing PA infections in healthcare settings.

## Methods

### Patient selection

During the period from January 2021 to December 2022, clinical isolates of PA were obtained from the Clinical Microbiology Laboratory of Guangdong Medical University Affiliated Hospital. Retrieve patient clinical data from the hospital’s electronic medical record system. The hospital primarily admits patients from the western region of Guangdong Province, China. Inclusion criteria: This study is limited to hospitalized patients. Outpatients or non-hospitalized individuals are not included. Patients must have a confirmed diagnosis of PA infection and present relevant clinical symptoms and signs to be eligible. Exclusion criteria: Patients with concurrent diseases that significantly impact infection characteristics and susceptibility test results are excluded, including severe hematological disorders, aplastic anemia, acute or chronic leukemia, as well as coexisting conditions like primary immunodeficiency and organ transplantation. Furthermore, patients who acquire both mPA and non-mPA simultaneously during a single hospital stay are also excluded. It should be specifically noted that if the patient tested positive for PA during multiple hospitalizations at different time periods, the statistical characteristics of the patient during each hospitalization need to be reassessed. If the isolates were collected on different days or if they can be distinguished based on antibiotic susceptibility, multiple isolates from the same patient are retained. This approach ensures a comprehensive evaluation of the patient’s characteristics and the variability of PA isolates over time.

Further data collection should include demographic characteristics (age and gender), duration of hospitalization, reason for hospitalization, concurrent infections, invasive procedures (such as Foley catheter, nasogastric tube, mechanical ventilation, tracheostomy, arterial and venous catheterization), underlying diseases (such as diabetes, hypertension), organ functional status (including hypoalbuminemia, septic shock, liver dysfunction, renal failure, heart failure, and respiratory failure), surgical history within the past year, history of steroid use (within the past 3 months), the levels of inflammatory biomarkers (within 3 days after the detection), the usage of antibiotics during the entire hospitalization period, and clinical clearance efficacy (effective, ineffective, unknown). The evaluation of the bacterial clearance rate is conducted by assessing the results of bacterial re-culturing during the hospitalization period.

### Susceptibility testing

Bacterial species identification and assessment of antimicrobial susceptibility were conducted using an automated Vitek-2 compact (bioMerieux, France). Phenotypic determination of mPA and non-mPA isolates was investigated by two phenotypic methods, Muir mordant staining and Congo red agar assay. The mucoid strains showed red colonies, and non-mucoid strains produced pink to white colonies on BHI agar containing Congo red and sucrose. In particular, the drug susceptibility testing for mPA was conducted employing the Kirby-Bauer disk diffusion method and interpreted according to the updated CLSI M100 Standard, utilizing PA ATCC 27853 as the quality control strain. The analysis of susceptibility test outcomes was processed utilizing WHONET 5.6 software (WHO, Geneva, Switzerland). The testing process determines the sensitivity of these drugs against specific bacterial strains. For instance, aminoglycosides like Amikacin, Gentamicin, and Tobramycin are examined to assess their effectiveness in combating target bacteria. Similarly, the susceptibility of carbapenems such as Imipenem and Meropenem, cephalosporins like Cefotetan, Cefepime, and Cefepime-Sulbactam, penicillins like Piperacillin and Piperacillin-Tazobactam, and quinolones like Ciprofloxacin and Levofloxacin are evaluated. Such testing aids in determining the efficacy of these agents against target bacteria and guides appropriate treatment strategies.

### Statistical methods

SPSS 26.0 Version (Chicago, IL, United States) was used for inferential analyses. Descriptive data is expressed in terms of values, rates, or mean (standard deviation). Different statistical methods, such as (adjusted) Chi-square test, Fisher’s exact test, T-test, and Mann–Whitney test, were employed to compare data, depending on the situation.

All tests were two-sided, and a value of *p* < 0.05 was considered significant. Effect estimates were expressed as odds ratio, with a 95% confidence interval.

## Results

### Clinical characteristics of the patients

**Figure 1:**
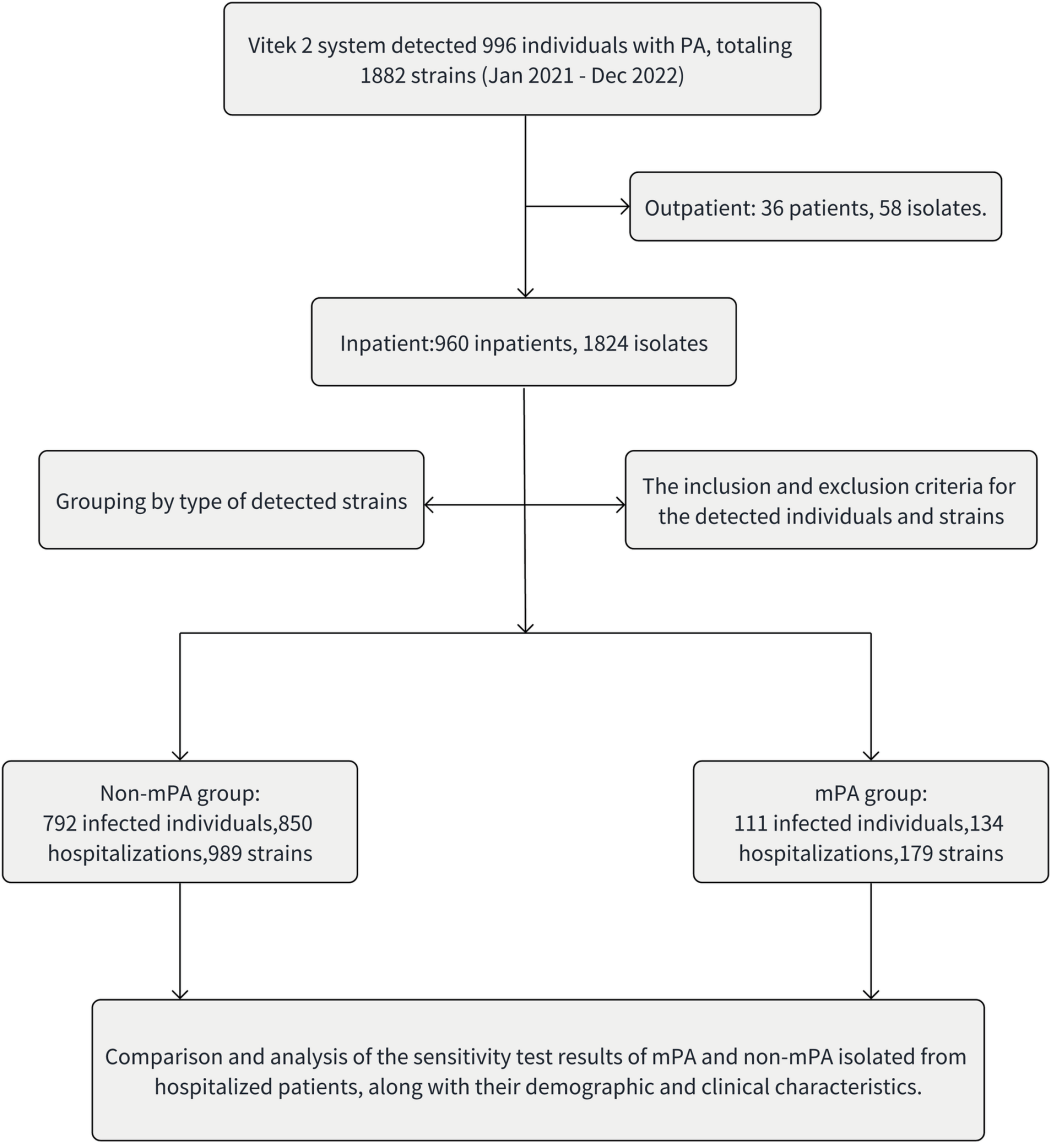
The flowchart shows essential statistical data on mPA and non-mPA in the study.

This study included a cohort of 111 patients diagnosed with mPA infections and 792 patients diagnosed with non-mPA infections. Additionally, 179 and 989 clinical isolates were, respectively, collected for the corresponding groups (Figure 1). The mPA group experienced a total of 134 hospitalization episodes, with 97 patients (87.4%) being hospitalized only once, while 14 patients (12.6%) had multiple hospitalizations (≥ two times). On the other hand, the non-mPA group had a cumulative frequency of 850 hospitalizations, with 747 patients (94.3%) being hospitalized only once, and 45 patients (5.7%) experiencing multiple hospitalizations. Significant differences were observed in the occurrence of repeated hospitalizations between patients with mPA and non-mPA infections, with a value of *p* of 0.006. Table 1 presents the clinical characteristics of patients afflicted with mPA and non-mPA infections.

**Table 1:** Characteristics of patients with mPA and non-mPA infections.

	mPA group	non-mPA group	*p* value
Age(years)	67.30 ± 11.73	61.91 ± 19.13	0.001
Gender			
Male	0.32(35/111)	0.67(530/792)	<0.001
Female	0.68(76/111)	0.33(262/792)	<0.001
Duration of hospitalization(days)	11.63 ± 6.47	25.93 ± 34.009	<0.001
Underlying diseases			
Diabetes	0.11(12/111)	0.19(149/792)	0.043
Hypertension	0.14(16/111)	0.36(288/792)	<0.001
Reason for hospitalization(top three)			
	Bronchiectasis (0.49; 65/134)	Cardiovascular and cerebrovascular disease (0.18; 155/850)	
	COPD (0.31; 41/134)	Trauma and fracture (0.16; 139/850)	
	Urinary calculus (0.04; 5/134)	Tumor (0.15; 126/850)	
Invasive procedures			
Foley catheter	0.19(26/134)	0.52(443/850)	<0.001
Nasogastric tube	0.11(15/134)	0.38(324/850)	<0.001
Mechanical ventilation	0.10(13/134)	0.40(339/850)	<0.001
Tracheostomy	0.01(1/134)	0.22(183/850)	<0.001
Arterial and venous catheterization	0.04(5/134)	0.25(214/850)	<0.001
Organ functional status			
Hypoalbuminemia	0.24(32/134)	0.61(518/850)	<0.001
Septic shock	0.05(7/134)	0.18(149/850)	<0.001
Liver dysfunction	0.07(10/134)	0.28(242/850)	<0.001
Renal failure	0.11(15/134)	0.27(231/850)	<0.001
Heart failure	0.24 (32/134)	0.28(240/850)	0.295
Respiratory failure	0.15(20/134)	0.41(351/850)	<0.001
History of steroid use (within the past 3 months)	0.54(72/134)	0.51(435/850)	0.582
Surgical history within the past year	0.04(6/134)	0.44(378/850)	<0.001
Concurrent infection	0.40(54/134)	0.59(501/850)	<0.001

In this study, the majority of hospitalized patients with mPA infections were found to have a single isolate within the past 2 years. However, among the 50 patients with mPA infections, two or more isolates were detected, and two patients had five or more isolates within 1 year. Among these isolates, the most common source was the lungs/airways, including samples such as sputum and bronchoalveolar lavage fluid, accounting for 92.2% (165/179) of the isolates. Other isolates were only found in urine and secretions, accounting for 8/179 (4.5%) and 6/179 (3.4%) of the total, respectively. On the other hand, non-mPA infections had a wider range of isolation sources. The primary source of isolates was still the lungs/airways, accounting for 53.8% (532/989) of the isolates. Other sources included wound secretions (24.5%, 242/989), urine (11.9%, 118/989), and peripheral blood (2.1%, 21/989), the others were also found in puncture fluid, drainage fluid, stool, bile, etc.

A summary of age and duration of illness history was conducted for the hospitalized patients who tested positive for mPA. It was found that these patients often had a long history of chronic respiratory system diseases. However, due to the lack of initial data on the presence of the bacteria, the initial infection and duration of the PA infection could not be determined at this time. Among the 34 hospitalized patients with mPA infection who had chronic obstructive pulmonary disease (COPD), 32 patients (94.1%) were in the acute exacerbation phase, while only 2 patients (5.9%) were in the stable phase. Among the 134 hospitalizations of mPA infections, 35.1% (47/134) of the patients were identified as having ineffective clearance. Effective treatment accounted for only 25.4% (34/134), while the majority of patients (53/134, 40.0%) underwent bacterial isolation and culture only during their first hospitalization, and it remains unclear regarding their clearance efficacy during that hospitalization. The subsequent treatment, bacterial culture, and identification have not been tracked. In contrast, the treatment effectiveness rate among non-mPA infection patients was 81.2% (690/850). Most patients infected with non-mPA can be successfully eradicated by appropriate antibiotic treatment.

### Indicators of inflammation in infected patients

To assess the host’s immune response after infection with mPA and non-mPA, inflammatory biomarker levels were measured during hospitalization. Table 2 illustrates the data pertaining to these inflammatory biomarkers for both the mPA and non-mPA infection cohorts.

**Table 2:** Inflammatory biomarkers in patients with mPA and non-mPA.

Inflammatory biomarkers	mPA group	non-mPA group	*p* value
White Blood Cell Count (x10^9/L)	8.89 ± 4.81	11.25 ± 6.92	<0.001
Neutrophil Count (x10^9/L)	6.58 ± 4.46	8.56 ± 4.85	<0.001
Lymphocyte Count (x10^9/L)	1.55 ± 0.73	2.07 ± 10.10	0.553
CRP (mg/L)	39.57 ± 47.85	77.50 ± 146.85	0.008
PCT (ng/mL)	1.06 ± 3.21	2.66 ± 9.47	0.252

### Susceptibility test

Due to the inability to guarantee complete susceptibility of the strains, unless otherwise specified, intermediate resistance is classified as resistance. Table 3 depicts the outcomes of susceptibility testing conducted on mPA and non-mPA strains.

**Table 3:** Results of antibiotic susceptibility testing for mPA and non-mPA.

	mPA group			non-mPA group		
Antibiotic	I (%)	R (%)	S (%)	I (%)	R (%)	S (%)
Amikacin	13(0.07)	22(0.12)	143(0.80)	9(0.01)	24(0.02)	956(0.97)
Ciprofloxacin	24(0.14)	50(0.28)	102(0.58)	61(0.06)	122(0.13)	788(0.81)
Meropenem	1(0.02)	7(0.11)	56(0.88)	31(0.03)	155(0.16)	766(0.80)
Piperacillin	4(0.09)	2(0.05)	37(0.86)	146(0.15)	98(0.10)	698(0.74)
Piperacillin-Tazobactam	21(0.12)	12(0.07)	143(0.81)	180(0.18)	47(0.05)	754(0.77)
Gentamicin	1(0.03)	–	31(0.97)	35(0.04)	33(0.03)	875(0.93)
Cefepime	14(0.08)	30(0.18)	124(0.74)	95(0.10)	66(0.07)	805(0.83)
Cefepime-Sulbactam	22(0.12)	11(0.06)	145(0.81)	148(0.18)	60(0.07)	620(0.75)
Cefotetan	4(0.02)	29(0.16)	143(0.81)	85(0.09)	111(0.11)	790(0.80)
Tobramycin	–	–	5(1.00)	15(0.02)	31(0.03)	907(0.95)
Imipenem	6(0.03)	40(0.22)	132(0.74)	29(0.03)	197(0.20)	761(0.77)
Levofloxacin	8(0.20)	9(0.22)	24(0.59)	56(0.06)	108(0.12)	815(0.83)

I, intermediate; R, resistant; S, sensitive; “–,” not detected.

Based on the susceptibility testing results, 29 strains (16.2%,29/179) of mPA were identified as CRPA. It is worth noting that 31.3% of the isolates (56/179) displayed sensitivity to all tested antimicrobial drugs. Additionally, among patients with multiple detections of mPA, a total of 20 patients (51.3%,20/39) exhibited inconsistent results in their drug susceptibility tests for mPA.

Within the cohort of patients diagnosed with COPD, a notable 17.6% (6/34) were found to be concurrently positive for CRPA. Likewise, among the group of individuals suffering from bronchiectasis, a corresponding 13.0% (7/54) were discovered to be positive for CRPA. It is worth highlighting that these patients were characterized by their advanced age, advanced-stage disease progression, and an extended duration of illness.

In contrast, among the strains of non-mPA, a total of 183 strains were identified as CRPA, accounting for 18.5% (183/989) of the tested strains. Additionally, amidst the cohort of 792 patients afflicted with non-mPA, incongruous outcomes were discerned in the drug susceptibility testing of 95 individuals (0.12, 95/792).

### Co-cultured microorganisms

To understand the clinical and microbiological correlations between PA and other microorganisms, we undertook an investigation to identify the co-isolated microorganisms within the same duration of hospitalization as PA.

Among the 134 hospitalizations analyzed, It was discovered that out of the patients infected with mPA, 39.4% (54 cases) did indeed experience co-infection with two or more bacterial pathogens. Fungi were the most frequently identified co-infecting microorganisms, comprising 61.1% (33/54) of the cases with secondary infections. Notably, *Candida albicans* was the predominant fungus identified, accounting for 78.8% (26/33) of these cases. However, it is important to consider that this finding may be region-specific. Staphylococcus species accounted for 11.1% (6/54) of the secondary infections, with the majority of cases involving methicillin-resistant *Staphylococcus aureus*, which accounted for 66.7% (4/6). In addition, *Stenotrophomonas maltophilia*, *Acinetobacter baumannii*, and *Klebsiella pneumoniae* comprised 9.3% (5/54), 9.3% (5/54), and 7.4% (4/54) of the cases, respectively.

In patients infected with non-mPA during a single hospitalization period, it was observed that 58.9% (501/850) had concurrent infections. The most prevalent coexisting pathogen detected was Enterobacteriaceae, which may be associated with a history of urinary catheterization, accounting for approximately 32.1% (161/501) of the total concurrent infections. The next commonly identified pathogens were *Acinetobacter baumannii*, *Klebsiella pneumoniae*, Fungi, *Staphylococcus aureus*, comprising 21.6%(108/501),16.4%(82/501),15.4%(77/501), and 15.0%(75/501)of the concurrent infections, respectively.

### Antibiotic utilization during hospitalization

To analyze the correlation between PA antibiotic susceptibility test results and drug usage, and investigate antibiotic consumption differences between the two groups. Table 4 displays the utilization of antibiotics throughout the hospitalization period.

**Table 4:** Antibiotic usage during hospitalization in patients with mPA and non-mPA.

Antibiotic	mPA group(%)	non-mPA group(%)	*p* value
Aminoglycosides	19(0.14)	76(0.09)	0.056
Quinolones	59(0.44)	382(0.45)	0.844
Beta-lactam antibiotics			
Penicillins	88(0.66)	607(0.71)	0.175
Cephalosporins	55(0.41)	467(0.55)	0.003
Carbapenems	29(0.22)	207(0.24)	0.495
Polypeptide			
Vancomycin	–	66(0.08)	–

## Discussion

PA is a prevalent bacterium accountable for nosocomial infections. It is capable of enduring on diverse surfaces and in the surrounding environment, and has the potential to induce severe acute and chronic infections among immunocompromised individuals. This often leads to a substantial extension of hospitalization periods and a heightened risk of mortality. Due to multiple factors, PA evades complete eradication within the host, resulting in persistent and long-term infections (4). Consequently, this paves the way for the adaptation of the bacterial pathogen to the host, facilitating the selection and accumulation of particular mutations in its genome. These mutations exert significant impacts on the pathogen’s overall physiology and toxicity (8, 10).

In the present study, patients with mPA infection are predominantly female, with a more concentrated age distribution. Their hospitalization reasons are also more limited, primarily related to respiratory system diseases such as bronchiectasis and chronic obstructive pulmonary disease. On the other hand, non-mPA infection patients exhibit distinct characteristics. Their hospitalization reasons are primarily associated with cardiovascular and cerebrovascular diseases, and closely related to patients with diabetes(*p* = 0.043) and hypertension(*p* < 0.001).

In terms of specimen sources, unlike acute infections caused by non-mPA, mPA infections typically do not involve bloodstream invasion and are usually confined to local infection sites. The sources of mPA isolates were more limited and have not yet been found in peripheral blood. In comparison, non-mPA, with its wider range of isolation sources including wound secretions, urine, and peripheral blood, bile, ascites and feces, etc. This may indicate a higher likelihood of systemic dissemination. Furthermore, in hospitalized patients with mPA, positive cultures for the bacterium are often obtained from clinical specimens collected within the first 3 days of admission. This early detection indicates a higher likelihood of mPA presence in these patients upon admission. On the other hand, when mPA is isolated from community-acquired cases, it is predominantly found in older adult patients with underlying comorbidities, particularly bronchiectasis and COPD. These comorbidities predispose individuals to chronic respiratory infections, and the presence of mPA may worsen the severity of the infection. Furthermore, observations indicate that mPA is more frequently detected in patients with a history of chronic respiratory diseases lasting over 10 years. These findings imply a potential correlation between mPA and specific diseases, along with a tendency for chronic and persistent infection. Consequently, for the prevention and treatment of mPA infections, it is crucial to consider and address these associated diseases, while also reinforcing monitoring, prevention, and treatment measures.

The study compared invasive procedures performed during hospitalization on patients infected with mPA and non-mPA. Foley catheterization, nasogastric tube insertion, mechanical ventilation, tracheostomy, arterial and venous catheterization showed statistically significant differences (*p* < 0.001). Additionally, patients with non-mPA exhibited more severe disease conditions with impaired organ function. These included hypoalbuminemia (*p* < 0.001), septic shock (*p* < 0.001), liver dysfunction (*p* < 0.001), renal failure (*p* < 0.001), and respiratory failure (*p* < 0.001). Compared to patients with mPA, those without the condition were found to have a higher occurrence of several risk factors, including undergoing surgery in the past year (*p* < 0.001), and having concurrent infection (*p* < 0.001). Such factors, in turn, raise the likelihood of infection in non-mPA patients, necessitating their careful consideration in the management of such infections. These results highlight the detrimental impact of non-mPA on patients’ clinical outcomes. Nevertheless, based on the data we have collected, it is important to acknowledge that patients with mPA infections often experience severe adverse clinical outcomes due to the presence of concurrent significant organic lesions. These organic lesions can further complicate the course of the infection and contribute to the development of more severe clinical symptoms. In an observational cohort study of 22,053 patients, the association between PA and the risk of mortality and disease exacerbation in patients with COPD was examined. The findings revealed a significant increase in the risk of disease exacerbation and mortality associated with PA infection (22). Furthermore, statistical analysis revealed significant differences in multiple inflammatory markers, specifically white blood cell count (*p* < 0.001), neutrophil count (*p* < 0.001), and CRP (*p* = 0.008). Our results indicate that non-mPA strains may lead to more severe inflammatory response during infection.

Upon reviewing the medical histories of 111 cases of mPA infection, it was discovered that even though the reasons for hospitalization were not directly tied to the detected infection sites, all patients exhibited symptoms and medical histories related to these sites. This indicates that the detection of mPA is not closely associated with the severity of the respective diseases related to its detection site. The bacteria coexist within the host without causing overwhelming damage (23). In clinical practice, PA infections are often classified as either “acute” or “chronic,” although the distinction between these categories is sometimes unclear. The ambiguity in clinical classification between acute and chronic may arise from the acute onset of various infections or underlying diseases. Acute PA infections typically manifest with symptoms in the lungs and are evaluated as ventilator-associated pneumonia (VAP) or community-acquired pneumonia (CAP). However, in certain conditions such as chronic bronchiectasis and COPD, PA can colonize the lungs without clear evidence of new disease episodes (24). In these patients, exacerbations of the chronic infections may clinically resemble new acute infections, leading to the misclassification of chronic infections as acute infections (25).

To address the issue of misclassification of chronic PA infections and reduce the incidence of acute exacerbations associated with bronchiectasis and COPD, it is important to implement appropriate prevention and treatment strategies. Additionally, gathering more information on the population structure and pathophysiological characteristics of PA infections in the context of bronchiectasis and COPD is crucial. This will help determine whether acute exacerbations during acute exacerbation of COPD (AE COPD) primarily represent true acute PA infections in the setting of chronic obstructive pulmonary disease or if they reflect acute exacerbations of the chronic infection process (25, 26).

Significant differences were observed in the susceptibility profiles of multiple antibiotics between the two bacterial strains (Table 2). Specifically, the mPA strain exhibited notably elevated levels of antibiotic resistance compared to the non-mPA strain. This increased resistance was particularly evident for amikacin (*p* < 0.001), ciprofloxacin (*p* < 0.001), cefepime (*p* = 0.003), and levofloxacin (*p* < 0.001). The results of bacterial susceptibility testing have revealed a correlation between antibiotic resistance and the frequent use of specific antibiotics. The extensive use of quinolones, penicillins, and cephalosporin antibiotics in our hospital has significantly contributed to the emergence of drug-resistant strains of PA against these specific drugs.

Clinical isolates of PA obtained from different patient groups have demonstrated varying antibiotic resistance levels, implying the existence of distinct subclonal populations. The presence of mixed bacterial communities can enhance the host’s resistance to antibiotics, exacerbate bacterial pathogenic adaptive mutations, and potentially compromise the ability to effectively clear the bacteria (27). The likelihood of treatment failures in infections caused by CRPA is rising due to the underappreciated existence of carba-penemases (9). A majority of patients with bronchiectasis and COPD, who are afflicted with carbapenem-resistant mPA, possess a substantial medical background. The recurrent identification of mPA strains in the same individual on multiple occasions emphasizes the difficulty in completely eliminating this particular strain. Respiratory tract infections caused by carbapenem-resistant mPA can further complicate the clinical management process.

In this study, the high-frequency detection of *Candida albicans* is considered an important co-pathogen in patients infected with PA. It has been demonstrated that PA can form mixed biofilms with *Candida albicans*, and the presence of both species in the biofilm enhances the accumulation of biomass compared to biofilms formed by a single species alone (28). These findings suggest that the presence of *Candida albicans* as a co-pathogen and the ability of PA to form mixed biofilms contribute to the persistence and resistance of mPA infections. The level of interaction between different microbial species within the biofilms also plays a role in determining the complexity of clinical diagnosis and treatment. This helps us understand how mixed bacterial communities impact antibiotic resistance, pathogenic adaptations, and bacterial clearance in the host (27, 29, 30).

Prior research has suggested that consistent identification of PA in sputum constitutes a notable predisposing factor for the development of mucoid conversion in PA. Individuals with compromised pulmonary function are particularly vulnerable to experiencing mucoid conversion of PA (31). When pulmonary function is impaired, the clearance efficacy of the respiratory tract may diminish, thereby heightening the likelihood of mucus accumulation and infections within the respiratory tract. This underscores the significance of lung function in both the prevention and control of PA infections (32). Inhibiting the occurrence of mucoid conversion in PA can effectively diminish its persistent survival within the host and mitigate its long-term consequences.

This study possesses several limitations. Firstly, it solely gathered information regarding antibiotic usage during the existing hospitalization for infection. There is a dearth of data pertaining to patient exposure to antimicrobial agents prior to the current hospitalization, a factor which may bear significant predictive value in relation to the occurrence of PA infection and drug resistance. Secondly, due to restricted bacterial culturing and identification, coupled with the absence of post-discharge follow-up on antimicrobial therapy for patients, a precise assessment of the effectiveness of eradication treatment for infections cannot be ascertained. Moreover, no distinction was made between co-infecting or coexisting microorganisms. Lastly, the specimens sampled merely represent a subset of the specimen source, suggesting that the detected microbial species and quantities are likely far more extensive and heterogeneous than what has been reported.

## Conclusion

In conclusion, our research has revealed that mPA possesses a distinctive ability to adapt to the host environment, which contributes to its comparatively milder clinical presentation. Nevertheless, the pronounced antibiotic resistance exhibited by these strains often poses a formidable challenge in achieving eradication, thereby culminating in chronic and persistent organ dysfunction for patients. Discrepancies in antibiotic resistance among diverse bacterial strains can serve as a crucial determinant for physicians to ensure the appropriate selection of antibiotics, consequently mitigating antibiotic misuse. In the case of mPA, their unique adhesion capabilities and ability to form biofilms may necessitate extended treatment durations and meticulous preventive measures. These discoveries are poised to offer invaluable guidance to physicians, enabling the development of effective clinical treatment plans and proactive strategies, thereby optimizing clinical practice.

## Data availability statement

The original contributions presented in the study are included in the article/supplementary material, further inquiries can be directed to the corresponding author.

## Ethics statement

The requirement of ethical approval was waived by Affiliated Hospital of Guangdong Medical University for the studies involving humans because As this was a retrospective study that solely analyzed pre-existing clinical and follow-up data, the requirement to obtain informed patient consent was waived. The studies were conducted in accordance with the local legislation and institutional requirements. Written informed consent for participation was not required from the participants or the participants’ legal guardians/next of kin because as this was a retrospective study that solely analyzed pre-existing clinical and follow-up data, the requirement to obtain informed patient consent was waived.

## Author contributions

ML: Data curation, Investigation, Writing – original draft. SL: Data curation, Writing – review & editing. WL: Project administration, Supervision, Writing – review & editing.
